# Genetics of hypertension: From experimental animals to humans

**DOI:** 10.1016/j.bbadis.2009.12.006

**Published:** 2010-12

**Authors:** Christian Delles, Martin W. McBride, Delyth Graham, Sandosh Padmanabhan, Anna F. Dominiczak

**Affiliations:** BHF Glasgow Cardiovascular Research Centre, Faculty of Medicine, University of Glasgow, UK

**Keywords:** GWAS, Genome-wide association study, QTL, Quantitative trait locus, SHR, Spontaneously hypertensive rat, SHRSP, Stroke-prone spontaneously hypertensive rat, SNP, Single nucleotide polymorphism, WTCCC, Wellcome Trust Case Control Consortium, Hypertension, Genetics, Rodents, Human

## Abstract

Essential hypertension affects 20 to 30% of the population worldwide and contributes significantly to cardiovascular mortality and morbidity. Heridability of blood pressure is around 15 to 40% but there are also substantial environmental factors affecting blood pressure variability. It is assumed that blood pressure is under the control of a large number of genes each of which has only relatively mild effects. It has therefore been difficult to discover the genes that contribute to blood pressure variation using traditional approaches including candidate gene studies and linkage studies. Animal models of hypertension, particularly in the rat, have led to the discovery of quantitative trait loci harbouring one or several hypertension related genes, but translation of these findings into human essential hypertension remains challenging. Recent development of genotyping technology made large scale genome-wide association studies possible. This approach and the study of monogenic forms of hypertension has led to the discovery of novel and robust candidate genes for human essential hypertension, many of which require functional analysis in experimental models.

## 1 Introduction

Hypertension is characterised by chronic elevation of blood pressure and affects 20 to 30% of the population worldwide [1]. Hypertension contributes significantly to the global burden of cardiovascular morbidity and mortality. A recent study by Lawes et al. [2] concluded that 13.5% of premature deaths and 54% of stroke and 47% of ischaemic heart disease worldwide are attributable to high blood pressure. Alarmingly, about 80% of the attributable burden occurred in low-income and middle-income economies, and over half occurred in people aged 45 to 69 years. Blood pressure is a continuous, quantitative trait whereas the dichotomous definition of hypertension (*i.e.* blood pressure above a certain threshold) is to some degree arbitrary and has been modified with expanding knowledge over the years. This has been acknowledged by recent guidelines where borderline or pre-hypertension states have been introduced and recommendations for blood pressure cut-offs and targets are dependent on comorbidities such as diabetes or renal disease [3,4]. Blood pressure in the upper range of normal is also associated with increased cardiovascular morbidity and mortality. Only half of the burden in the study by Lawes et al. [2] was in people with hypertension, whereas the remainder was in subjects with lesser degrees of high blood pressure (≥ 115 mmHg but < 140 mmHg systolic). It appears that there is a continuous relationship between blood pressure and cardiovascular risk without evidence of a threshold down to at least 115/75 mm Hg [5].

These considerations have major implications on studies into the genetics of hypertension. A qualitative trait of “hypertension” could be subject to classic Mendelian laws of inheritance whereas a continuous “blood pressure” trait would constitute a non-Mendelian complex trait [6]. The normal unimodal distribution of blood pressure in the general population supports the latter [7]. However, there are rare monogenic Mendelian forms of hypertension which demonstrate that at least some forms of hypertension and possibly even some components of blood pressure could be explained by classic Mendelian inheritance [6]. From family and twin studies heritabilities of systolic and diastolic blood pressure are generally estimated in the range of 15 to 40% and 15 to 30%, respectively [8–10]. The sibling recurrent risk of hypertension is in the range of 1.2 to 1.5 [11]. However, these figures are influenced by non genetic factors including shared environment and measurement errors so that the magnitude of the genetic effect could be different.

Another fundamental problem in the genetics of hypertension is the definition of the ancestral phenotype. Hypertension is a disease of modern civilisation and heavily depends on environmental and particularly dietary factors of modern society. For example, members of the Luo tribe had lower blood pressure in their traditional rural environment than in the urban environment of Nairobi where their urinary sodium concentration was higher and urinary potassium concentration was lower [12]. It has been hypothesised that in a sodium-deprived environment the default genotype is a sodium conserving one. Likewise, the renin–angiotensin–aldosterone system may have initially been adapted for sodium conservation but may play an important role in the pathogenesis of hypertension in modern societies with high dietary salt intake [13]. These considerations highlight that for genes associated with hypertension the disease allele does not necessarily have to be the minor (rare) allele but could be the major (for examples sodium conserving) allele as well, and that studies into genes associated with low blood pressure could be equally important for the genetics of hypertension.

Our attempts to unravel the genetic basis of human essential hypertension are complicated by the above considerations and other challenges we will refer to later. We have previously reviewed this topic [14] and concluded that a multifaceted approach including and integrating human and animal studies is a most promising approach. Ten years later we take the opportunity to update our previous review. There has been major progress in some areas, mainly driven by the advances in technology and statistics, but it is fair to say that we are still not much closer to a complete understanding of the genetics of hypertension than a decade ago.

## 2 The rat as a physiological model of hypertension

One strategy to overcome the limitations of functional genetic studies in humans is to take advantage of specific hypertensive rat strains that have been selectively bred over many generations and are inherently simpler paradigms, but remain under complex control [15,16]. This is especially relevant, as recent developments in genome sequencing [17] and software development [18,19] have accelerated conserved genome analysis and translational studies between rat and human. Numerous genome-wide studies in rat models have revealed candidate quantitative trait locus (QTL) regions on almost every rat chromosome, as well as some important interactions between loci [15,20]. As with humans, however, the critical rate-determining step is no longer the identification of QTLs, but the identification of the genetic determinant underlying the QTL.

### 2.1 Quantitative trait loci influencing blood pressure

The principal strategy in the rat for the search of genes involved in the development of hypertension has been the identification of QTLs responsible for blood pressure regulation by genome-wide scanning [15,21]. The difficulties in identifying QTLs include epistasis, and a limited statistical power due to the number of hypotheses being tested. Moreover, any single QTL is responsible for only a fraction of the phenotypic variation and thus the phenotype–genotype correlation is low. Improvements in genomic resources and statistical analysis have led to the identification of a large number of QTLs. However, despite the numerous complex traits that have been analysed by genome-wide linkage studies, few of the underlying genes have been identified [22].

**Fig. 1:**
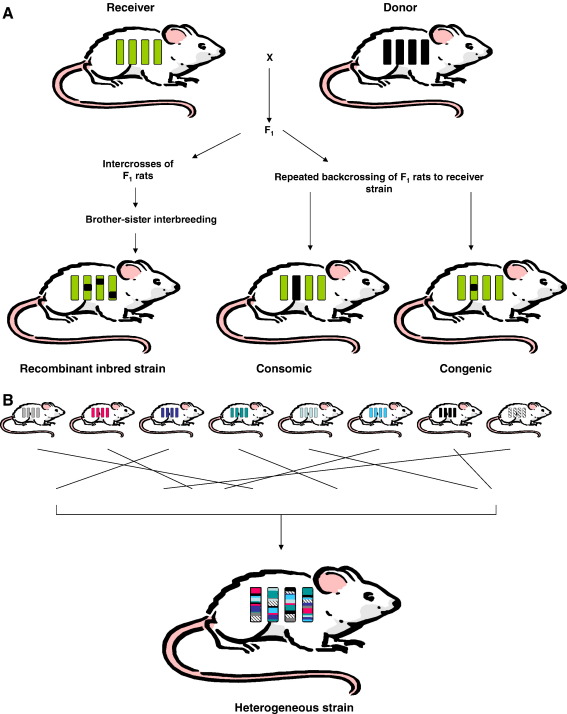
Selected analytical tools in rat genetics. A) The construction of recombinant inbred animals which are genetic mosaics of the two founding strains, consomic (chromosome substitution), congenic strains. B) An alternative method for fine-mapping small-effect QTLs uses outbred rats of known ancestry, genetically heterogeneous stock (HS). HS rats are generated by intercrossing several inbred progenitor strains followed by cross breeding for more than 50 generations.

QTL mapping is a phenotype driven approach that does not require prior knowledge of either causative genes or their function and can lead to the identification of novel genes involved in disease. Furthermore, in animal models there is also the distinct advantage of the construction of congenic strains to first verify the existence of the QTL, and also to allow production of congenic substrains to enable genetic dissection and fine-mapping in order to reduce QTL intervals (Fig. 1). This congenic strategy was considered ideal, since initial estimates suggested the involvement of a relatively few major effect genes, thus requiring the construction of essentially Mendelian-like strains to facilitate gene identification. However, recent congenic studies have demonstrated the underlying genetic complexity of large QTL regions identified in genome-wide scans. Comprehensive congenic coverage on various rat chromosomes has revealed multiple QTLs, with evidence for loci with opposing effects on blood pressure [23–28]. While congenic fine-mapping can restrict the number of genes in a QTL interval to a minimum to facilitate gene identification [29], frequent loss of the hypertensive phenotype suggests the existence of multiple genes. Furthermore, potential epistatic interactions (*i.e.* the masking of a gene by the effect of another unrelated gene) may underlie many QTL peaks and congenic intervals [30,31], and this has hampered progress towards the identification of causative genetic elements.

## 3 Identifying genetic determinants

Combining congenic fine-mapping, comparative genomic tools, gene expression array resources, transgenic rescue and knockout technology in the rat provides a powerful tool for the discovery of novel genes underlying complex cardiovascular traits. The first successful example of this combined strategy was the identification of the gene encoding Cd36 as a major contributor to insulin resistance and dyslipidaemia in the spontaneously hypertensive rat (SHR) [32], confirmed by transgenic rescue [33] and subsequent translation to human populations [34,35]. However, there was an issue concerning the contribution of *Cd36* to hypertension. These issues have only recently been resolved by implicating *Cd36* as a determinant of blood pressure and risk for hypertension by specifically identifying deficient renal expression of *Cd36* [36].

Our own research has focused on the stroke-prone spontaneously hypertensive rat (SHRSP), where the genetic determination of hypertension is due to multiple gene–gene and gene–environment interactions. Although exceptionally high blood pressure is clearly a factor for end-organ damage in the SHRSP, genetic factors are also likely to contribute. In particular, two blood pressure QTLs have been mapped to rat chromosome 2 [37]. This region of chromosome 2 is a classic example of a common or overlapping QTL because it has been implicated in several rat crosses. These two QTLs, therefore, have become a focus for further congenic strategies in our laboratory [38] and other laboratories [39]. In combination with congenic strain construction in the SP.WKYGla2a and SP.WKYGla2c* strains, genome-wide microarray expression profiling identified glutathione S-transferase mu type 1 (*Gstm1*) as a positional candidate gene for spontaneous hypertension [40–42], and endothelial differentiation gene 1 (*Edg1*) and vascular cell adhesion molecule 1 (*Vcam1*) for salt sensitivity [43].

It is notable that there are still relatively few examples where this strategy has led to the identification of positional and functional hypertension candidate genes that can be translated and tested in human populations [44,45]. It is also important to note that narrowing the QTL interval by substitution mapping remains a critical part of this combined strategy, as demonstrated recently by the subsequent elimination of the differentially expressed candidate gene, *Resp18*, with the production of the smallest reported rat blood pressure QTL interval of 117 kb [46]. This highlights that, even with the onset of extensive genome sequencing, our ability to identify a full complement of genes and other genetic elements continues to be a challenge. However, recent evidence suggests that once again, this locus is more complex than originally believed as further transgenic–congenic rats confirm the eligibility of *Resp18* as a hypertension candidate gene [47]. This highlights the requirement to modulate the levels of target genes in the appropriate genetic background with either transgenic over expression or knockout rats by embryo microinjection of zinc-finger nucleases [48].

It should be noted, however, that the efficiency of candidate gene identification is very much dependent on the complexity of the phenotype under investigation. There are many examples of other disease phenotypes in the rat that have proved more amenable to candidate gene discovery via congenic strategies including pristane-induced arthritis [49] and cresentric glomerulonephritis [50,51].

## 4 Consomic, heterogeneous rat and recombinant inbred strains

As well as advances in genome resources there are a number of large scale animal resources available for exploitation (Fig. 1).

Consomic rat strains are generated by introgressing an entire chromosome from one inbred strain into the isogenic background of another inbred strain using marker-assisted selection. The PhysGen (http://pga.mcw.edu) Program for Genomic Application at the Medical College of Wisconsin developed two panels of consomic rats using the SS/JrHsdMcwi, the FHH/EurMcwi and the BN/NHsdMcwi strains. Characterization of these consomic strains, each carrying a chromosome from the sequenced Brown Norway strain, allows for immediate mapping of traits to a particular chromosome without the need for genetic crosses. This is ongoing work, but gene expression [52] and proteomic [53] analysis has been undertaken in a number of these consomic strains. From the consomic strains, congenic strains can be rapidly bred within 6 months to narrow the region on a specific chromosome to a region that can be targeted for gene identification [54].

A further strategy to improve the efficiency of gene identification is to utilise the rat heterogeneous (HS) stock panels. HS rats are generated by intercrossing several inbred progenitor strains followed by cross breeding for more than 50 generations. Each chromosome is a fine-grained mosaic of the progenitor strains: the average distance between recombinants is small (less than 2 cM) so that the HS provide high resolution mapping of multiple QTLs across the genome. This allows fine-mapping of QTLs to sub-centimorgan intervals by exploiting recombinants that have accumulated over many generations of out breeding in genetically HS that are derived from inbred strains (Fig. 1). This significantly reduces the number of candidate genes found within each QTL. The best characterised rat heterogeneous stock panel was established at the NIH in 1984 and derives from eight genetically distinct and phenotypically diverse inbred strains (ACI/N, BN/SsN, BUF/N, F344/N, M520/N, MR/N, WKY/N and WN/N). Pilot studies in 786 Rat HS [55] strains support the findings from the mouse HS [56] indicating refinement of a behaviour QTL identification from 40 cM to 13 Mbp.

One of the great successes in the rat over the past few years has been the exploitation of the rat recombinant inbred panel (Fig. 1). A comprehensive genetic screen of the rat recombinant inbred strains has recently been undertaken in which gene expression values were considered as expression quantitative traits [57]. This integration of genome-wide expression profiling with linkage analysis is a new approach to identifying genetic determinants underlying complex traits. The expression profiling of kidney and fat RNA from all individuals allows the profile of each gene, treated as an intermediate phenotype, to be considered as an expression quantitative trait. Furthermore, by conserved genome analysis, a data set of 73 candidate genes for hypertension, including *Cd36* and *Gstm1,* have been identified that merit translational studies in human populations [57]. Other notable successes with this strategy include candidate genes for heart failure [58] and left ventricular hypertrophy [59].

## 5 Translation from animal to man

In our own studies in the SHRSP we have identified the glutathione S-transferase mu type 1 gene (*Gstm1*) as a positional candidate for hypertension in the SHRSP [41]. Glutathione S-transferases are involved in the defences against oxidative stress and thereby also constitute functional candidates for hypertension [60]. We and others have confirmed reduced *Gstm1* gene [41,57,61] and Gstm1 protein expression [42] in SHRSP. The human orthologues of *Gstm1* are therefore promising candidate genes for essential hypertension. However, when we performed a definite association study of *GSTM* genes in hypertension involving sequencing of *GSTM* genes, genotyping for the *GSTM1* deletion and studies in three different and independent cohorts, we were unable to show a significant association between any of the human *GSTM* genes and hypertension [62].

This apparently negative example illustrates that direct translation of findings in experimental animal models to human disease is not without challenges. To the best of our knowledge there are no examples of a direct translation of findings in rodents to man for the genetics of hypertension. Whilst this may in part be due to differences in the genetics of hypertension between animals and humans it also illustrates the general challenges we are facing in candidate gene studies of human essential hypertension.

## 6 Candidate gene studies

Candidate genes are chosen for their assumed role in the pathogenesis of hypertension and can derive from experimental models or knowledge of the pathophysiology. The most plausible candidate genes are components of the renin–angiotensin–aldosterone system and genes involved in signal transduction (*e.g.* the G-protein subunit-β3 gene *GNB3*), salt and water handling (*e.g.* the adducin-α genes *ADD1*), regulation of vascular tone (*e.g.* the endothelial nitric oxide synthase gene *NOS3*) and production of or defences against oxidative stress. However, despite numerous reports on association in small and medium sized cohorts since publication of our previous review [14] there has been little evidence for a substantial association of any of these genes or single nucleotide polymorphisms (SNPs) within these genes with hypertension. The main reasons for this lack of positive results are (1) the individual contribution of genetic variants to the blood pressure phenotype is small; (2) most of the study designs lacked power to detect these small effects; (3) inconsistency of phenotyping did not allow results to be compared or combined; (4) selection of candidate genes depended on known pathways of blood pressure regulation precluding undiscovered pathways; (5) most of the candidate gene studies tended to be single SNP studies which was not completely informative of the gene variations and additional SNP identification needed sequencing resources which were not readily accessible; (6) studies of rare frequency variants were not done; and (7) the study design issues mentioned above precluded any meaningful study of gene–gene or gene–environment interactions. Nevertheless, the candidate gene approach remains one of the cornerstones of studies into the genetics of hypertension. We refer to recent reviews on the genetics of the renin–angiotensin–aldosterone system [63–65], G-proteins [66], adducin [67], and oxidative stress [68,69] in hypertension, but will discuss the association of the aldosterone synthase gene (*CYP11B2*) with hypertension as an exemplar.

The physiology of aldosterone is relatively well understood. There is considerable epidemiological evidence that aldosterone has a role in the development of hypertension and other cardiovascular diseases. Even within the physiological range, increased plasma aldosterone levels predispose to development of hypertension [70]. Increased aldosterone levels in adulthood may be determined by conditions early in life such as foetal growth [71]. While plasma aldosterone and aldosterone to renin ratio were found to be heritable phenotypes, common polymorphisms in the CYP11B2 gene explained only partially variations in aldosterone levels [72,73]. This is akin to the previous experience with ACE and AGT levels and like before a large number of studies have been performed in recent years and these were meta-analysed by Sookoian et al. [74]. The evidence from the meta-analysis was borderline for association between hypertension (qualitative trait) and the C-344T variant of *CYP11B2* and no association seen with systolic and diastolic blood pressure (quantitative trait). In any meta-analysis, the results depend on the constituent studies, and this is true of most of the studies included here and all the reasons mentioned above are applicable. Like other candidate genes, aldosterone synthase is an attractive target, and it is likely that evidence for association may come from studies of rare or structural variants in this locus.

With the advances of technology and reduced costs for genotyping we will see more large scale candidate gene projects in the future. An example for this approach is the ITMAT/Broad/CARE Vascular Disease 50 k SNP Array (IBC Chip; http://bmic.upenn.edu/cvdsnp/). This chip allows genotyping of about 50,000 SNPs in 2100 cardiovascular candidate genes at relatively low costs. Studies into hypertension have been conducted but reports are still outstanding. The relatively large scale of these projects necessitates correction for multiple tests and will therefore have an unfavourable impact on sample size and power of such studies. Extracting gene specific data from genome-wide SNP chips are not very fruitful. A recent example of a study into 160 candidate genes for blood pressure covering 2411 SNPs in 1644 subjects of the KORA S3 cohort is a case in point [75]. None of the SNPs remained significantly associated with blood pressure after Bonferroni correction. The top twelve hits in seven genes for association with blood pressure and/or hypertension (*P* < 10^− 3^) failed to be replicated in independent cohorts.

## 7 Linkage studies

In contrast to candidate gene studies we have seen significant developments in the area of genome-wide linkage and association studies since publication of our previous review [14]. Historically, linkage studies were the first technically feasible genome-wide studies of human hypertension. Linkage studies examine transmission of disease loci from parents to offspring, but modifications of this concept allowed studies complex traits in the form of affected sib-pair studies [76]. The latter is a powerful method of performing linkage studies with small family sizes, but there are practical issues in recruitment of families with a mobile adult population worldwide, although we have recently demonstrated that family-based recruitment is still possible [77].

The British Genetics of Hypertension (BRIGHT) study is probably the best example for a linkage study into essential hypertension [11]. The consortium genotyped 2,010 affected sibling pairs drawn from 1599 severely hypertensive families, and completed a 10 cM genome-wide scan. Linkage analysis identified a principle locus on chromosome 6q, with a lod score of 3.21 that attained genome-wide significance (*P* = 0.042). Three further loci with lod scores higher than 1.57 (2q, 5q, and 9q) also showed genome-wide significance (*P* = 0.017) when assessed under a locus-counting analysis. Genes or functional genetic variants underlying the linkage peaks have still not been identified. The authors originally interpreted these results as evidence for an oligogenic element of human essential hypertension. However, insufficient marker density and insufficient sample size to detect genetic variants with small effects on blood pressure were thought to be limitations of the BRIGHT study. These limitations have recently been addressed by regenotyping hypertensive probands from the BRIGHT study as part of SNP based genome-wide association studies (GWAS) into common disease [78].

There have been a number of similar genome-wide linkage studies. QTLs for blood pressure have been found on virtually all human chromosomes in linkage studies [79]. Koivukoski et al. [80] meta-analysed 9 genome-wide scans of blood pressure or hypertension using the genome-search meta-analysis method and found susceptibility loci on chromosomes 2 (2p12–q22.1) and 3 (3p14.1–q12.3). Their study demonstrated that sample size is a crucial factor detection of markers with genome-wide significance. Subsequently, we demonstrated a successful attempt at reducing heterogeneity by using antihypertensive drug response to partition different pathways of hypertension [81]. In the BRIGHT population, hypertensive sib-pairs who were non-responsive to ACE inhibitors, ARBs or beta-blockers showed significant linkage on chromosome 2p (LOD = 4.84 at 90.68 Kosambi cM). This susceptibility locus co-localises to a region found in African-American hypertensives in the Family Blood Pressure Program who showed evidence of linkage with hypertension status at 93 cM with a LOD score of 2.84 [82]. Thus the chromosomal 2p locus independently identified in different populations may contain a gene or genes for the salt-sensitive form of hypertension which is common among Africans, and the same mechanism may be operative in a subset of white European hypertensives identified by unresponsiveness to beta-blockers and ACE inhibitors. Another approach was taken by the BRIGHT investigators who performed linkage analysis of covariate data including serum and urine biochemistry, biometric measurements and additional blood pressure phenotypes [83]. Genome-wide significant results were obtained for body mass and renal function related traits on chromosomes 20 and 5, respectively. Although these results need to be interpreted with caution as the analysis has not been adjusted for treatment and was performed in a selected population of hypertensive subjects, additional phenotypic information may help to reduce genetic heterogeneity and identify novel loci for hypertension.

## 8 Genome-wide association studies

Linkage studies are extremely attractive tools in human genetics but they can only identify highly penetrant loci and have been notably successful in the identification of monogenic forms of hypertension, The recent developments in genotyping technology with the introduction of chip-based genotyping arrays [84,85] has paved the way for GWAS that genotype for a large number of genetic markers usually SNPs (for example about 500,000 in the Wellcome Trust Case Control Consortium (WTCCC) study [78]) as opposed to the highly polymorphic microsatellite markers that have been used in classic genome-wide linkage studies. With the resources from Hapmap, it is possible to impute up to 2.5 million SNPs in LD with the tag SNPs present in these GWAS SNP arrays.

In 2007 two major GWAS into hypertension have been reported. Levy et al. [86] conducted a study in Framingham Heart Study families and associated blood pressures at two different time points and long-term averaged blood pressure with 100,000 polymorphic markers using the Affymetrix 100 k chip. In the primary analyses, none of the associations attained genome-wide significance. The other study was reported by the WTCCC and examined association with 500,000 genetic markers in 2000 patients with hypertension (derived from the BRIGHT cohort) and 3000 control subjects using the Affymetrix 500 k chip [78]. Also the WTCCC study did not find a SNP for hypertension that achieved genome-wide statistical significance. At the same time, the WTCCC examined other common diseases against the same common control subjects and successfully identified loci for coronary artery disease, bipolar disorder, rheumatoid arthritis, Crohn's disease and type 1 and 2 diabetes. Whilst the WTCCC study was characterised by better marker density compared to the Framingham Heart Study, there have been a number of criticisms about the study design that may have caused underperformance of hypertension compared to the other common diseases in the WTCCC study. The common controls were not selected and in part not even phenotyped for blood pressure. With a prevalence of hypertension of 20 to 30% in the general population [1] is very likely that the presence of cases in apparent controls has reduced the power to identify disease specific associations [78]. This concern has been partly addressed by the selection of cases from the top 5% of blood pressure distribution in the UK population thereby reducing significantly the number of misclassified controls with the same phenotype as the cases. Nevertheless, due to the results of these two GWAS there has been disappointment and concern that the effect sizes of genes related to hypertension could be lower than those observed for other common diseases where odds ratios in the range of 1.2 to 1.5 were found [87]. Most recently a study in 8842 samples from a Korean general population with replication in 7861 independent samples revealed an association of rs17249754 near the *ATP2B1* gene at genome-wide significance level [88]. The effect size of this SNP was relatively small (− 1.309 ± 0.266 mmHg for systolic and − 0.882 ± 0.181 mmHg for diastolic blood pressure), but certainly in a realistic range for a multigenic disorder.

The major breakthrough came with the report of two studies that examined associations of a large number of SNPs with blood pressure and hypertension. The Global BPgen consortium tested 2.5 million genotyped or imputed SNPs for association with systolic or diastolic blood pressure in 34,433 subjects of European ancestry [89] and identified eight regions with genome-wide significance. The variants were near the *CYP17A1*, *CYP1A2*, *FGF5*, *SH2B3*, *MTHFR*, *ZNF652* and *PLCD3* genes and chromosome 10 open reading frame 107 (c10orf107). These loci were also associated with hypertension as qualitative trait. The second consortium (CHARGE) studied 2.5 million genotyped or imputed SNPs in 29,136 subjects and found significant (*P* < 4 × 10^− 7^) associations with systolic blood pressure for 13 SNPs, with diastolic blood pressure for 20 SNPs and with hypertension for 10 SNPs [90]. The data were than meta-analysed with those of the Global BPGen consortium [89] and revealed genome-wide significance (*P* < 5 × 10^− 8^) for *ATP2B1* (chromosome 12q21; plasma membrane calcium ATPase 1), *CYP17A1* (chromosome 10q24; steroid 17-alpha-monooxygenase), *PLEKHA7* (chromosome 11p15; pleckstrin homology domain containing, family A member 7), *SH2B3* (chromosome 12q24; SH2B adaptor protein 3), *CACNB2* (chromosome 10p12; calcium channel, voltage-dependent, beta 2 subunit), *CSK-ULK3* (chromosome 15q24; adjacent to c-src tyrosine kinase and unc-51-like kinase 3 loci), *TBX3-TBX5*, (chromosome 12q24; adjacent to T-box transcription factor TBX3 and T-box transcription factor TBX5 loci) and *ULK4* (chromosome 3p22; unc-51-like kinase 4) for association with systolic or diastolic blood pressure or hypertension.

Despite the impressive samples sizes and even more impressive *P*-values it should be noted that the effects of these SNPs on blood pressure are rather modest. For example, in Global BPgen the SNP showing the strongest association with systolic blood pressure (rs11191548, *P* = 7 × 10^− 24^) increases systolic blood pressure by 1.16 mmHg per major allele. Generally an effect of about 1 mmHg per identified SNP can be assumed which may have major implications for hypertension related cardiovascular disease in the population but minor implications for an individual. Nevertheless, these projects have clearly demonstrated the existence of blood pressure susceptibility genes with small but measurable effects. Most of the SNPs are located in genes that have not immediately been thought to play a major role in the pathogenesis of hypertension, and it is hoped that these findings will improve our understanding of the underlying pathomechanisms.

## 9 Monogenic forms of hypertension

Since publication of our review in 2000 [14] there has been considerable progress in the area of rare Mendelian forms of hypertension. These syndromes account for less than 1% of human hypertension and because a clear and often treatable reason has been elucidated, do not immediately explain the genetics of essential hypertension. However, the Mendelian forms of hypertension are important because they constitute the archetype of hereditable hypertension; demonstrate the importance of genes expressed in the kidney and responsible for salt and water handling in the pathogenesis of hypertension; and may help to unravel mechanisms that also apply to the pathogenesis of essential hypertension. Mendelian forms of hypertension have been reviewed recently [6,79,91] and are summarised in Table 1. It should be noted that there are additional syndromes that would go beyond the scope of this review. For instance, loss-of-function mutations in the aldosterone synthase gene (CYP11B2) can lead to reduced aldosterone levels, hypovolaemia, hypotension and shock [92–94]. Other examples include familiar hyperaldosteronism type II, congenital adrenal hyperplasia, mutations in the *PPARG* gene and hypertension and bradydactyly [6,79,91]. Some of these syndromes can be caused by mutations in several genes, some have not yet been localised to a gene, or have been localised but still lack understanding of the mechanisms that lead to hypertension.

One of the major discoveries in recent years was the description of the WNK (with no K [lysine]) kinases and their role in the pathogenesis of hypertension. The WNK1 and WNK4 serine-threonine kinases regulate the sodium, chloride and potassium transport mechanisms in the distal nephron [95–97]. *WNK1* and *WNK4* mutations have been identified to cause Gordon syndrome (pseudohypoaldosteronism type 2) [98]. It has then be demonstrated that variants of the *WNK1* gene not only cause this severe Mendelian form of hypertension but that more subtle variants are also associated with blood pressure variation in a severely hypertensive [99] and in the general population [100].

This finding supports the hypothesis that common variants underlying the Mendelian forms of hypertension could affect blood pressure variation in the general population and thereby be involved in the pathogenesis of hypertension. In 2008, Tobin et al. [101] demonstrated that variants of the *KCNJ1* gene causing Bartter syndrome type 2 (Table 1) were strongly associated with mean 24-hour systolic or diastolic blood pressure. Associations were also found for *CASR*, *NR3C2*, *SCNN1B* and *SCMM1G* (Table 1). Although this study was a milestone in understanding the role of common variants in genes causing monogenic hypertension for regulation of blood pressure in the general population it indeed dealt with common variants (minor allele frequency > 1%). The study by Tobin et al. [101] is a candidate gene study and awaits replication in independent cohorts. In the same year, however, Ji et al. [102] opened a completely new avenue of research by also testing three genes which underlie Mendelian forms of hypertension (*SLC12A3*, *SLC12A1* and *KCNJ1*) in a general population cohort derived from the Framingham Heart Study. In contrast to the work by Tobin et al. [101], these authors have screened all coding exons and flanking introns for rare DNA sequence variants and found a total of 138 different coding sequence variants in 2492 subjects [102]. These mutations were associated with reduced prevalence of hypertension. Quantitatively, mean systolic blood pressure was 5.7 mmHg, 6.4 mmHg and 9.0 mmHg lower at the age of 40, 50 and 60 years, respectively, in carriers of the mutation compared to non-carriers. Thereby, these rare mutations in just three genes contributed equally or more to blood pressure variation in the general population than the common variants identified by the recent GWAS [89,90]. Direct sequencing may be a promising tool to identify rare (*i.e.* minor allele frequency < 1%) mutations that are not usually covered in GWAS. The above example demonstrates that such rare variants in genes whose pathophysiological role in the regulation of blood pressure is beyond any doubt can help to explain blood pressure variability in the population.

## 10 Summary and open questions

As a result of enormous effort a number of plausible and validated genetic variants that are associated with blood pressure and/or hypertension have recently been found. It emerged that these variants are either common and in turn contribute little to blood pressure variation [89,90] or are rare and have a relatively strong effect [102]. The future development of this area is difficult to predict but will most likely see a combination of GWAS and sequencing studies in large cohorts.

The past ten years have also demonstrated that accurate phenotyping is one of the keys to discover genetic determinants of disease. This may be less of an issue in relatively clearly defined diseases such as type 1 diabetes or Crohn's disease but is a major problem in hypertension where there is a somewhat arbitrary cut-off for definition of disease, marked sexual dimorphism, marked age effect, and a substantial influence of environmental factors. Unsurprisingly genetics of hypertension has been less of a success story compared to other common diseases, but the high impact of hypertension on cardiovascular morbidity and mortality more than justifies further efforts. The role of intermediate phenotypes including endothelial function and vascular stiffness but also associated phenotypes such as body weight and renal function [83] in gene discovery remains to be elucidated. It is also not clear whether an approach in the general population targeted towards blood pressure variation (quantitative trait) or an approach in selected extremes of cases and controls (quantitative trait) is superior. Most likely both approaches have their own merits.

Finally, we have witnessed substantial progress in both human genetics and animal models of hypertension, but have seen few truly translational studies. It appears that both approaches have been developed in parallel with little focus on informing each other. This will almost certainly change in the next few years, but probably not necessarily in the direction “animal to man” but more likely in the direction “man to animal”. The highly sophisticated tools that are now available for human genomic studies have led to the identification of numerous causative genes and genetic loci that require detailed studies of function in experimental models.

## Figures and Tables

**Table 1:** Mendelian forms of hypotension and hypertension.

Disorder	Gene/locus	Age of onset	Disease mechanism	Features	References
*Syndromes associated with low blood pressure*					
Gitelman syndrome	*SLC12A3*	Adolescence or adulthood	Loss-of-function mutations in the gene encoding the thiazide-sensitive Na–Cl cotransporter of the distal collecting duct causes salt wasting and activation of the renin–angiotensin–aldosterone system. Thereby maintenance of serum sodium but loss of potassium and H^+^ by augmentation of the epithelial sodium channel	Hypokalaemia with metabolic alkalosis. Low serum Mg^2+^ and low urinary Ca^2+^ levels	[103,104]
	16q3				
Bartter syndrome				Often associated with preterm delivery. In contrast to Gitelman syndrome, normal or only mildly reduced serum Mg^2+^ and increased urinary Ca^2+^ levels	
Type 1	*SLC12A*	Neonatal	Loss of function of the apical Na–K–2Cl cotransporter		[105]
	15q21				
Type 2	*KCNJ1*	Neonatal	Mutations of the ATP-sensitive K^+^ channel ROMK affect K^+^ recycling and thereby inhibit Na^+^ reabsorption in the thick ascending limb of Henle		[106]
	11q24				
Type 3	*CLCNKB*	School age	Loss-of-function mutations in the Cl^−^ channel CLCNKB in the thick ascending limb of Henle. Some of these mutations have arisen from unequal crossing over between *CLCNKB* and the nearby related gene *CLCNKA*		[107]
	1p36				
Type 4	*BSND*	Neonatal	*BSND* encodes Barttin which functions as a beta-subunit for CLCNKA and CLCNKB chloride channels	Associated with sensorineural deafness. inhibition of NaCl reabsorption in type IV Bartter syndrome is not restricted to the thick ascending limb of Henle	[108]
	1p32				
Type 5	*CASR*	Adulthood	Mutations activating the calcium-sensing receptor CaSR which then inhibits sodium transport in the thick ascending limb of the loop of Henle	Associated with autosomal dominant hypocalcaemia (ADH)	[109]
	3q13				
Autosomal dominant pseudohypoaldosteronism type I	*NR3C2*	Neonatal	Loos/of/function mutations in the mineralocorticoid receptor impairing maximum salt absorption. The reduced activity of the endothelial sodium channel affects H^+^ and K^+^ excretion	Salt wasting with hypotension despite markedly elevated aldosterone levels; hyperkalemia and metabolic acidosis. Often asymptomatic in adulthood on usual western (high salt) diet	[110,111]
	4q31				
Recessive pseudohypoaldosteronism type I	*SCNN1A*	Neonatal	Loss-of-function mutations in any of the three different subunits of the epithelial sodium channel	Salt wasting and hypotension with hyperkalemia and metabolic acidosis, despite high levels of aldosterone. Require high dose salt supplementation	[112]
	16p12				
	*SCNN1B*				
	12p13				
	*SCNN1G*				
	16p12				

*Syndromes associated with high blood pressure*					
Glucorticoid-remediable aldosteronism	*CYP11B2* and *CYP11B1*	Second or third decade	Gene duplication due to unequal crossing over between the aldosterone synthase (*CYP11B2*) and steroid 11β-hydroxylase (*CYP11B1*) genes. The resulting chimaeric gene encodes a protein under the regulation of ACTH with aldosterone synthase activity	Normal or elevated aldosterone levels despite suppressed plasma renin activity. Hypokalaemia and metabolic alkalosis are variable associated findings. Exogenous glucocorticoids completely suppress aldosterone secretion	[113–116]
	8q21				
Apparent mineralocorticoid excess	*HSD11B2*	Childhood	Absence of the enzyme 11β-hydroxysteroid dehydrogenase allows cortisol to activate MR, resulting in hypertension mediated by increased epithelial sodium channel activity	Hypokalaemia and metabolic alkalosis accompanied by suppressed plasma renin activity and the virtual absence of circulating aldosterone	[117,118]
	16q22				
Hypertension exacerbated in pregnancy	*NR3C2*	Before second decade	Missense mutation, S810L, in the mineralocorticoid receptor causing normal activation by aldosterone but also activation by ligands that are normally silent or antagonistic (*e.g.* progesterone)	Exacerbation during pregnancy due to the increased progesterone levels	[119]
	4q31				
Liddle syndrome	*SCNN1B*	Adolescence	Mutations in either the β or the γ subunit of the epithelial sodium channel delete their cytoplasmic C termini and result in increased channel activity	Associated with hypokalaemic alkalosis, suppressed plasma renin activity, and low plasma aldosterone levels	[120–122]
	12p13				
	*SCNN1G*				
	16p12				
Pseudohyperaldosteronism type II (Gordon syndrome)	*WNK1*, 12p13	Second or third decade	WNK kinases are expressed in the distal nephron and are involved in the control of renal electrolyte homeostasis	Hyperkalaemia and low aldosterone levels. Sensitive to treatment with thiazide diuretics	[95,98]
	*WNK4*, 17q21				
